# Predictors of Hungry Bone Syndrome and Reintervention After Subtotal Versus Total Parathyroidectomy for Secondary Hyperparathyroidism in Dialysis Patients: A Single-Center Cohort

**DOI:** 10.3390/jcm14144944

**Published:** 2025-07-12

**Authors:** Adina Coman, Cristi Tarta, Gigi Adrian Aiordachioae, Dan Goldis, Diana Utu, Marco Marian, Amadeus Dobrescu, Florina Buleu, Sorin Olariu

**Affiliations:** 1 Researching Future Surgery II Research Center, Department X, Discipline of General Surgery II, Faculty of Medicine, Victor Babes University of Medicine and Pharmacy Timisoara, E. Murgu Square, No. 2, 300041 Timisoara, Romania; adina.coman@umft.ro (A.C.); marian.marco@umft.ro (M.M.); dobrescu.amadeus@umft.ro (A.D.); 2 Doctoral School, Victor Babes University of Medicine and Pharmacy Timisoara, E. Murgu Square, No. 2, 300041 Timisoara, Romania; 3 Department X, Discipline of General Surgery I, Faculty of Medicine, Victor Babes University of Medicine and Pharmacy Timisoara, E. Murgu Square, No. 2, 300041 Timisoara, Romania; olariu.sorin@umft.ro; 4 Department of Medicine, Discipline of Surgery II, Vasile Goldiş Western University, Liviu Rebreanu Boulevard, No. 86, 310414 Arad, Romania; aiordachioaie.gigi@uvvg.ro (G.A.A.); goldis.dan@uvvg.ro (D.G.); 5 Department II, Discipline of Pharmacology-Pharmacotherapy, Faculty of Pharmacy, Victor Babes University of Medicine and Pharmacy Timisoara, E. Murgu Square, No. 2, 300041 Timisoara, Romania; diana.utu@umft.ro; 6 Department VI, Discipline of Internal Medicine and Ambulatory Care, Prevention and Cardiovascular Recovery, Faculty of Medicine, Victor Babes University of Medicine and Pharmacy Timisoara, E. Murgu Square, No. 2, 300041 Timisoara, Romania; florina.buleu@umft.ro

**Keywords:** parathyroid glands, secondary hyperparathyroidism (SHPT), chronic kidney disease–mineral bone disorder (CKD-MBD), total versus subtotal parathyroidectomy, persistent and recurrent hyperparathyroidism, hungry bone syndrome, thymectomy, predictive risk factors for postoperative outcomes, alkaline phosphatase

## Abstract

**Background/Objectives**: Secondary hyperparathyroidism (SHPT) is a prevalent complication in end-stage renal disease, often necessitating surgical intervention when refractory to medical therapy. The optimal surgical strategy—subtotal parathyroidectomy (SPTX) versus total parathyroidectomy with/without autotransplantation (TPTX ± AT)—remains debated, especially considering postoperative complications like persistent HPT and hungry bone syndrome (HBS). This study aimed to compare early surgical outcomes and identify predictors for postoperative complications in patients undergoing SPTX and TPTX + AT. **Methods**: We conducted a retrospective, single-center observational study involving 93 dialysis patients who underwent PTX for drug-refractory SHPT. Patients were analyzed according to surgical procedure (SPTX vs. TPTX + AT), focusing on postoperative complications such as cervical bleeding, reintervention rates, and the incidence of HBS. Multivariate logistic regression was utilized to identify predictors of these outcomes. **Results**: TPTX + AT demonstrated superior control of HPT, with significantly lower rates of reintervention compared to SPTX (7.1% vs. 23.5%, *p* = 0.037). However, TPTX + AT was associated with a higher incidence of HBS (57.1% vs. 35.3%, *p* = 0.039). Independent predictors of reintervention included absence of concomitant thymectomy, preoperative hypercalcemia, fewer visualized glands preoperatively, and preoperative PTH > 2000 pg/mL. Elevated alkaline phosphatase levels (>300 U/L), severe bone pain, and the TPTX procedure itself were significant predictors of HBS. **Conclusions**: Surgical strategy for SHPT should be individualized, balancing the lower recurrence risk associated with TPTX + AT against its higher likelihood of postoperative hypocalcemia. Preoperative biochemical markers and clinical features could potentially influence operative decision-making and optimize patient outcomes.

## 1. Introduction

Secondary hyperparathyroidism (SHPT) is a hallmark complication of end-stage renal disease (ESRD) characterized by excessive parathyroid hormone (PTH) secretion and parathyroid gland hyperplasia. In chronic kidney disease (CKD), phosphate excretion is impaired, leading to chronic phosphate retention and hyperphosphatemia. Elevated serum phosphorus, along with 1,25-dihydroxyvitamin D (calcitriol) deficiency (due to loss of renal 1α-hydroxylase activity) and resultant hypocalcemia, triggers increased PTH synthesis and parathyroid cell proliferation. Over time, diffuse polyclonal hyperplasia can transition to nodular hyperplasia of the glands, with nodules of monoclonal cells that grow autonomously and become refractory to medical therapy [1].

SHPT emerges as an adaptive physiological response to multiple mineral disturbances that trigger and sustain elevated PTH secretion through both traditional mineral imbalances and recently identified hormonal disruptions. The classical pathogenic mechanisms underlying SHP development prominently feature dysregulation of phosphate, calcium, and calcitriol homeostasis. Beyond these classical drivers, contemporary research has identified additional mechanisms in SHPT pathophysiology. Notably, excess fibroblast growth factor-23 (FGF23) coupled with reduced Klotho, as well as skeletal resistance to PTH due to disrupted Wnt–β-catenin signaling (exacerbated by uremia-related intestinal dysbiosis), have all been implicated in maintaining hyperparathyroidism in CKD [2].

This modern understanding highlights the complex, multifactorial nature of SHPT [2]. Together, these pathogenic factors cause persistently elevated PTH levels in most dialysis patients unless therapeutic measures are taken. The chronically elevated PTH in SHPT leads to a cascade of mineral and bone disorders, as well as systemic complications, known collectively as CKD–mineral bone disorder, namely: high-turnover renal osteodystrophy, intractable bone pain and fractures, extensive vascular/valvular calcifications, refractory anemia and, ultimately, increased cardiovascular and all-cause mortality [3].

Contemporary medical therapy—dietary phosphate restriction, calcium- or non-calcium-based phosphate binders, active vitamin-D analogues, and calcimimetic agents—may achieve biochemical control and attenuate SHPT progression in many patients. However, achieving target PTH levels (2–9× upper limit of normal in dialysis patients [4]) often proves to be quite challenging in clinical practice. It is estimated that ~15% of patients on dialysis for 10 years, and up to ~38% after 20 years, will ultimately require parathyroidectomy (PTX) due to severe, drug-refractory SHPT [5]. International guidelines therefore recommend surgery once markedly elevated PTH persists, despite optimized pharmacological therapy. PTX was traditionally recommended at PTH levels >800 pg/mL [6]. However, more recent clinical guideline updates emphasize using an individualized threshold, highlighting surgery as the definitive option when medical measures fail [7]. Conversely, PTX has been shown to improve hypertension control, anemia (erythropoietin resistance), bone mineral density, fracture rates, and even survival in this population [8].

To this day, two distinct operative strategies dominate SHPT surgical management in clinical practice: subtotal PTX (SPTX), wherein ≈3½ glands are excised, leaving a remnant (<100 mg) in situ, to preserve endogenous PTH and mitigate permanent hypocalcemia; and total PTX with autotransplantation (TPTX + AT), which removes all cervical parathyroid tissue, but then reimplants a small parathyroid tissue specimens—usually in the forearm—in order to minimize recurrence, while also maintaining some PTH reserve [9]. In addition, some centers advocate TPTX without AT, i.e., TPTX alone, often combined with a cervical thymectomy to excise any supernumerary parathyroid tissue. This approach removes all parathyroid tissue without reimplantation, virtually eliminating recurrence risk but rendering the patient permanently dependent on exogenous calcium/vitamin D supplementation [10].

Even though these approaches tend to rapidly normalize calcium-phosphate metabolism, each carries distinctive associated risks. Herein, insufficient resection during SPTX may permit hyperplastic regrowth of the remnant, leading to persistent or recurrent SHPT, often requiring reintervention for additional surgical resection of parathyroid tissues, whereas TPTX, especially when performed without AT, is more prone to prolonged/permanent hypocalcemia and hungry bone syndrome (HBS)—reported in up to 70% of high-turnover cases [9]. HBS, a well-recognized complication, entails severe, prolonged hypocalcemia with acute skeletal uptake of calcium following PTX. HBS can manifest as life-threatening tetany or arrhythmia if not aggressively managed with calcium and calcitriol supplementation [11].

Evidence directly comparing the two PTX approaches is mixed and remains debatable. For the most part, the retrospective studies and meta-analyses available tend to indicate that both procedures have comparable efficacy in reducing PTH and relieving symptoms, with no significant differences in overall survival or recurrence rates [12,13]. However, more recent analyses have highlighted differences. A 2017 systematic review found that TPTX without AT significantly lowers the rates of recurrent or persistent hyperparathyroidism compared to TPTX + AT [10], albeit with a higher risk of transient postoperative hypoparathyroidism [10]. On the other hand, a 2020 network meta-analysis (incorporating all three surgical options) concluded that TPTX + AT offers the best balance of efficacy and safety, and recommended it as the preferred surgical approach for SHPT [14].

Conversely, a 2019 meta-analysis of >3600 patients found similar rates of residual disease and symptom improvement between SPTX and TPTX + AT [12]. A Swedish nationwide registry study observed that, as opposed to SPTX, TPTX significantly reduced the risk of repeat parathyroid surgery (adjusted hazard ratio ~0.30), but was also associated with a higher incidence of cardiovascular events, while overall survival and fracture rates were, again, comparable [15]. TPTX with a forearm autograft offers the distinct advantage that any recurrence can be managed by percutaneous excision of the graft under local anesthesia, avoiding a repeat cervical exploration, necessary in SPTX to remove the orthotopic hyperfunctioning remnant [16].

High-level evidence from randomized clinical trials (RCTs) is limited. Notably, an early RCT by Rothmund et al. (1991) compared SPTX versus TPTX + AT in 40 patients, finding that TPTX + AT achieved more complete remission of hyperparathyroidism with no reoperations needed, whereas SPTX led to recurrent disease requiring reoperation in several cases [17]. The largest multi-center RCT in the field contrasted TPTX alone with TPTX + AT and therefore did not resolve this controversy [18]. Consequently, operative choice still hinges on surgeon preference, institutional tradition, and individual patient factors. The dilemma is further complicated in kidney transplant recipients, where persistent or “tertiary” hyperparathyroidism affects 20–50% of patients and jeopardizes graft survival. Surgical strategies in the transplant population must also consider technical aspects of graft protection and long-term functional outcomes, as highlighted in recent evaluations of post-transplant surgical planning and biomarker development [19,20]. Recent comparative data suggest that PTX confers better allograft outcomes than continued cinacalcet therapy in this setting [21]. Thus, clarifying which surgical approach offers the optimal balance between durable PTH control and postoperative complications is timely.

Against this background, we conducted a retrospective single-center observational study comparing early surgical outcomes of SPTX versus TPTX + AT in drug-refractory SHPT. We specifically examined cervical bleeding requiring re-exploration, persistent or recurrent hyperparathyroidism mandating re-intervention, and the incidence of hungry bone syndrome, while adjusting for patient age, dialysis vintage, and biochemical disease severity. Our aim is to provide evidence that may refine operative decision-making and improve the multidisciplinary management of severe SHPT in the modern calcimimetic era.

## 2. Materials and Methods

### 2.1. Study Design and Patient Selection

We conducted a retrospective single-center observational study of ESRD patients on dialysis, with SHPT, who underwent PTX, performed within the Department of General Surgery II, at Timisoara County Emergency Clinical Hospital, between January 2016 and December 2021. All surgical records within this timeframe for parathyroid surgery were retrospectively reviewed according to the relevant procedural codes by two surgeons (A.C. and M.M.) and independently verified by an attending senior surgeon (C.T.). All study procedures were approved by the Research Ethics Committee of the Victor Babes University of Medicine and Pharmacy Timisoara (31/2015/rev2022).

The study inclusion criteria were adults (≥18 years), undergoing renal replacement therapy (hemodialysis or peritoneal dialysis), with biochemically manifest SHPT (e.g., PTH persistently >800 pg/mL with hypercalcemia and/or hyperphosphatemia), refractory to maximal medical therapy (phosphate binders, calcitriol/vitamin D analogs, and calcimimetics). In our institution, SHPT patients were referred for surgical consultation by nephrologists, exclusively following medical treatment failure. The surgeons were not involved in the primary nephrological care or initial medical management of these ESRD patients. Herein, all patients were required not only to have undergone either a subtotal or total PTX, as indicated by the surgical team, based on gland morphology and surgeon preference, but also to have regularly visited our in-house nephrology clinic for check-ups and periodical follow-up. Exclusion criteria included tertiary HPT (post-renal transplantation), primary HPT, parathyroid malignancy, or incomplete records of outcomes.

A final study cohort of 93 patients met the inclusion criteria, of whom 51 underwent SPTX and 42 underwent TPTX. SPTX was defined as resection of 3 to 3½ glands, leaving a remnant of approximately 50–100 mg of the most normal-appearing parathyroid tissue in situ. TPTX was defined as resection of all identified parathyroid tissue; in 26 of 42 TPTX cases (61.9%), a small fragment of parathyroid tissue was autotransplanted into the forearm. In the remaining 16 TPTX cases, no AT was performed, effectively rendering the patient aparathyroid (these were typically older patients or those for whom recurrence prevention was paramount). Cervical thymectomy was performed in 15 patients (all in the TPTX group) to remove potential ectopic parathyroid tissue within the thymus. All surgeries were performed by experienced endocrine surgeons; intraoperative PTH monitoring was not routinely used. Informed consent was obtained for surgery in all cases, as dictated by routine clinical practice.

### 2.2. Data Collection

Patient demographics, relevant comorbidities (diabetes, vascular disease), and baseline biochemical parameters were recorded, including age, sex, dialysis vintage (years on dialysis), preoperative serum creatinine, calcium (albumin-corrected), phosphate, alkaline phosphatase (ALP), and intact (i) PTH levels. Clinical features of SHPT were noted (muscle weakness/fatigability, bone pain, pruritus, ectopic calcifications, etc.). We categorized bone pain severity using a Visual Analogue Scale (VAS), as reported in the admission charts: mild (VAS 1–3), moderate (4–6), or severe (7–10). Radiological evidence of renal osteodystrophy such as osteopenia/osteoporosis or calciphylaxis was documented if present. Osteoporosis and osteopenia were defined in accordance with internationally accepted criteria (World Health Organization and International Society for Clinical Densitometry guidelines) based on bone mineral density assessment by dual-energy X-ray absorptiometry (DEXA) using a Hologic Discovery A densitometer (Hologic Inc., Bedford, MA, USA). Specifically, osteoporosis was defined as a T-score ≤ −2.5, and osteopenia as a T-score between −1.0 and −2.5. The presence of urinary calculi was also noted. Furthermore, preoperative evaluation of the parathyroid glands, namely, the number of glands reported as enlarged/hypertrophic, was also analyzed and documented for each case.

Operative details were assessed to confirm the type of PTX, use of AT, and adjunct procedures (thymectomy). Intraoperative details (number of glands identified, weight of resected tissue) and immediate postoperative course were reviewed. Postoperative nadir calcium and iPTH levels during hospitalization for PTX (within 1 week after surgery) were recorded. In addition, we determined the proportion of patients whose early postoperative iPTH fell below two times the assay’s upper limit of normal (ULN). While this threshold has traditionally been used as a target, recent evidence suggests that very low PTH levels may increase the risk of adynamic bone disease. In our cohort, 71 of 93 patients (76.3%) achieved an iPTH below 2× ULN. Notably, this occurred significantly more frequently in the TPTX group (37/42, 88.1%) compared to the SPTX group (34/51, 66.7%, *p* = 0.016), reflecting the more complete parathyroid tissue removal with TPTX. Patients were closely monitored and managed as per standard protocol with oral/IV calcium and calcitriol to mitigate hypocalcemia.

Key outcomes of interest were defined as follows:

1. *Early local bleeding*: cervical hematoma post-PTX, requiring surgical exploration, evacuation, and hemostasis, within 48 h of the initial surgery. This typically presented as anterior cervical tumefaction, with or without respiratory distress.
2. *Reintervention for additional parathyroid tissue excision due to inadequate PTH control:* any surgical reintervention for either persistent HPT (i.e., iPTH > 300 pg/mL, immediately after surgery, likely indicating retained hyperactive parathyroid tissue); or recurrent HPT (i.e., iPHT < 300 pg/mL within 6 months from primary PTX, but gradually increasing to exceed this threshold again after 6 months, usually accompanied by associated clinical symptoms). Persistence was confirmed by imaging and biochemistry, and reintervention usually took place during the index hospitalization or soon after.
3. *Hungry bone syndrome (HBS)*: a syndrome of profound, prolonged hypocalcemia after surgery, operationally defined in our center as corrected serum calcium <8.0 mg/dL (2.0 mmol/L) for >4 days post-PTX despite supplementation, accompanied by hypophosphatemia and/or elevated ALP, consistent with increased bone uptake. All patients with HBS will require high-dose calcium infusion and calcitriol therapy.

Follow-up data were obtained for a median of 4.2 years (interquartile range 3.1–5.6 years) after PTX. Only four patients were lost during long-term follow-up, due to mortality of unrelated causes. For patients who had recurrent HPT, details of any secondary interventions (medications or reoperation) were noted.

We retrospectively collected operative time (defined as the interval between incision and closure recorded in surgical notes) and postoperative hospital length of stay (LOS, defined as the total number of days from surgery until discharge).

### 2.3. Statistical Analysis

All analyses were performed in IBM SPSS Statistics (v29, IBM Corp, Armonk, NY, USA), with supplementary graphics generated in GraphPad Prism (v10.1, GraphPad Software, San Diego, CA, USA). Continuous variables were first checked for normality by the Shapiro–Wilk test and inspection of Q–Q plots; they are therefore reported as mean ± standard deviation when normally distributed and compared with two-tailed Student’s *t*-tests (using Welch’s correction if Levene’s test indicated unequal variances), or as median and inter-quartile range when skewed and compared with Mann–Whitney U tests. Categorical data are presented as counts and percentages and evaluated with χ^2^ tests or Fisher’s exact tests when expected cell counts were <5, while ordered categories such as VAS pain grades were assessed for linear trend with the Cochran–Armitage test.

The study had two co-primary outcomes: (i) surgical reintervention for persistent or recurrent hyperparathyroidism and (ii) hungry bone syndrome (HBS). For each, potential predictors were first screened in univariable analyses; variables achieving *p* < 0.20, together with age, sex, and surgical approach (pre-specified for clinical relevance), were entered into multivariable logistic-regression models that were refined by backward stepwise selection guided by the Akaike information criterion. Adjusted odds ratios are reported with 95% confidence intervals, model calibration was verified with the Hosmer–Lemeshow test, and discrimination was quantified by the area under the receiver operating characteristic curve (AUC). To guard against over-fitting, coefficients were optimism-adjusted by 1000-sample bootstrap resampling. Dose–response relationships between biochemical markers (iPTH, ALP) and outcomes were further explored with restricted cubic-spline terms, and a pragmatic bedside risk-score for reintervention was derived directly from model β-coefficients and internally validated (10-fold cross-validation, c-statistic, and calibration-in-the-large). Pearson or Spearman correlations, as appropriate, were then assembled into a correlation matrix to visualize inter-relations between key continuous variables and outcomes, while a decision algorithm was generated by recursive-partitioning (rpart), pruned with the 1-SE rule.

Because two co-primary endpoints were specified a priori, a Bonferroni-adjusted significance threshold of α = 0.025 was applied to their hypothesis tests; all other analyses used two-sided α = 0.05. For exploratory correlation analyses involving multiple variables, we applied a Bonferroni correction to control for multiple comparisons, considering correlations significant only if *p* < 0.01. Missingness was <5 % for every variable, permitting complete-case analysis; nevertheless, multiple imputation by chained equations (five datasets) yielded materially identical estimates. The statistical workflow adhered to STROBE guidance for observational cohorts and contemporary best-practice recommendations for prognostic-model development and internal validation.

## 3. Results

### 3.1. Patient Demographics and Baseline Characteristics

A total of 93 patients (mean age 53.1 ± 11.5 years; 60% male) underwent PTX for drug-refractory SHPT. Detailed baseline characteristics of patients undergoing SPTX (*n* = 51) and TPTX (*n* = 42) are presented in Table 1. Patients selected for TPTX were significantly older, with a mean age of 56.8 years compared to 50.2 years in the SPTX group (*p* = 0.01), and demonstrated a notably longer median dialysis vintage (8.1 years versus 7.0 years, albeit not reaching statistical significance—*p* = 0.18).

Approximately 40% of patients in each group had diabetes, and 25% had a history of cardiovascular disease (coronary or cerebrovascular). All patients had been treated with conventional SHPT therapies: all were on non-calcium phosphate binders and active vitamin D; 60% of TPTX patients and 57% of SPTX patients had been on cinacalcet (a calcimimetic) for at least 6 months before surgery (*p* = 0.79). Despite medical therapy, PTH and mineral levels remained markedly abnormal.

The clinical presentation of advanced skeletal disease was comparable between surgical groups. In fact, manifestations of advanced SHPT were prevalent across the study population. Moderate-to-severe bone pain (VAS 4–10) affected the majority of patients in both groups (80.9% of TPTX patients and 76.5% of SPTX patients), with no significant differences in the prevalence of renal lithiasis, osteoporosis, osteopenia, or pathological bone fractures, although trends suggested more severe disease manifestations among TPTX patients. Systemic manifestations were similarly distributed between surgical approaches, with refractory pruritus reported in approximately 65% of patients, muscular weakness and fatigability in 64.6% of the cohort (66.7% TPTX vs. 62.7% SPTX, *p* = 0.86), and calcific uremic arteriolopathy (calciphylaxis) documented in a small subset of patients (3 patients, all in the TPTX group). Approximately one-third (32%) of the cohort presented with marked hypercalcemia (>11 mg/dL), indicative of autonomous parathyroid function approaching tertiary HPT, with similar distribution between surgical groups. These characteristics underscore that all patients had advanced, multiglandular SHPT unresponsive to medical management, meeting accepted indications for surgery.

Preoperative biochemical parameters indicated higher disease severity in the TPTX group, characterized by significantly elevated median iPTH (2084 pg/mL vs. 1570 pg/mL, *p* = 0.01) and ALP (281.5 U/L vs. 240.3 U/L, *p* = 0.05). Preoperative serum calcium levels were slightly higher in the TPTX group (10.7 vs. 10.2 mg/dL, *p* = 0.03).

Preoperative localization studies were performed in all patients (ultrasound, computed tomography, and/or magnetic resonance imaging)—unfortunately, 99mTc-sestamibi scintigraphy and positron emission tomography were unavailable regionally for the better part of the study timeframe. These were primarily to assist surgical planning and did not dictate the choice of subtotal vs. total resection. The number of enlarged glands identified on preoperative imaging showed no significant variation between PTX subgroups, but this was often limited by nodular thyroid disease or ectopic glands (one patient had an undescended parathyroid in the carotid sheath found at surgery).

### 3.2. Surgical Specifics and Primary Postoperative Outcomes

All surgeries were completed under general anesthesia without intraoperative complications. The mean weight of excised parathyroid tissue was substantial (total of 3.8 ± 1.0 g in TPTX patients, and 3.5 ± 0.9 g in SPTX patients, *p* = 0.22), reflecting significant glandular hyperplasia in both groups. In SPTX cases, a viable remnant was left in situ in the cervical compartment. Concomitant AT was exclusively performed in patients undergoing TPTX (61.9% vs. 0%, *p* < 0.001), consistent with the nature of the procedural approach, i.e., forearm autografts generally utilized 8–12 small fragments of a hyperplastic gland implanted in the brachioradialis muscle. Similarly, concomitant thymectomy was also performed solely in the TPTX group (35.7% vs. 0%, *p* < 0.001).

Early postoperative complications, specifically local bleeding requiring surgical exploration, were infrequent, occurring in 5 out of 93 patients (5.4%) overall, with no statistically significant difference between surgical groups (TPTX: 7.1% vs. SPTX: 3.9%, *p* = 0.82). All five cases of postoperative hemorrhage were managed by prompt exploration and hemostasis with no long-term sequelae; none had undergone thymectomy, and there was no clear pattern by surgical approach. Thus, the risk of post-op cervical bleeding appeared low and similar for both procedures.

**Figure 1:**
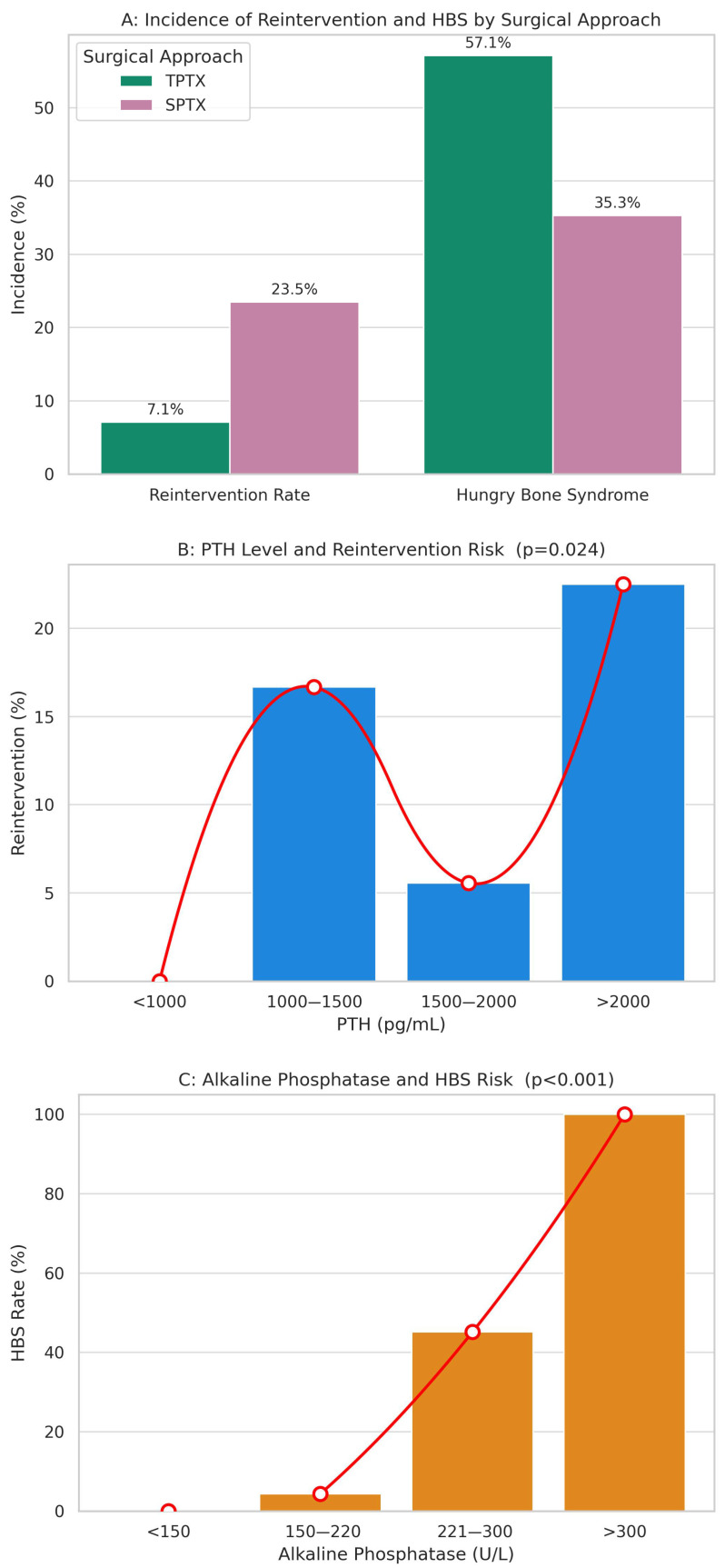
Primary Outcomes and Risk Relationships: (**A**) Comparison of reintervention and hungry bone syndrome (HBS) incidence following total parathyroidectomy (TPTX) versus subtotal parathyroidectomy (SPTX). TPTX was associated with a significantly lower reintervention rate (7.1% vs. 23.5%, *p* = 0.064), but a higher incidence of HBS (57.1% vs. 35.3%, *p* = 0.058) compared to SPTX; (**B**) Distribution of reintervention rates by preoperative parathyroid hormone (PTH) levels. A progressive increase in reintervention is seen with higher PTH categories, peaking at >2000 pg/mL (22.5%, *p* = 0.024), consistent with a dose–response effect; (**C**) HBS incidence increases sharply with rising serum ALP levels. While no cases of HBS occurred with ALP <150 U/L, rates escalated to 100% for values >300 U/L (*p* < 0.001), demonstrating a strong correlation between bone turnover markers and postoperative calcium demands.

Refractory HPT requiring additional surgical intervention was significantly more frequent after SPTX. Figure 1A highlights this essential difference between the two approaches, with 12 of 51 SPTX patients (23.5%) requiring reintervention, compared to only 3 of 42 TPTX patients (7.1%), a difference that also achieved statistical significance (*p* = 0.037). In the SPTX failures, all cases involved hyperplastic remnants, with several patients having extremely high pre-op PTH (>2000 pg/mL), suggesting that subtotal resection was insufficient for such severe disease burden. Conversely, the TPTX failures were all in patients who had not undergone concomitant thymectomy, with imaging eventually revealing an ectopic fifth gland in two cases and incomplete resection in the third. This finding underscores the importance of thymectomy during TPTX to remove potential ectopic parathyroid tissue.

The data demonstrates a clear trade-off between surgical approaches. While TPTX offered superior protection against persistent/recurrent disease, it was associated with a higher incidence of HBS compared to SPTX (57.1% vs. 35.3%, *p* = 0.039). All patients who underwent a second surgery, regardless of initial approach, achieved definitive biochemical remission of SHPT thereafter, confirming that proper identification and complete removal of all hyperactive parathyroid tissue is ultimately achievable in this patient population.

**Figure 2:**
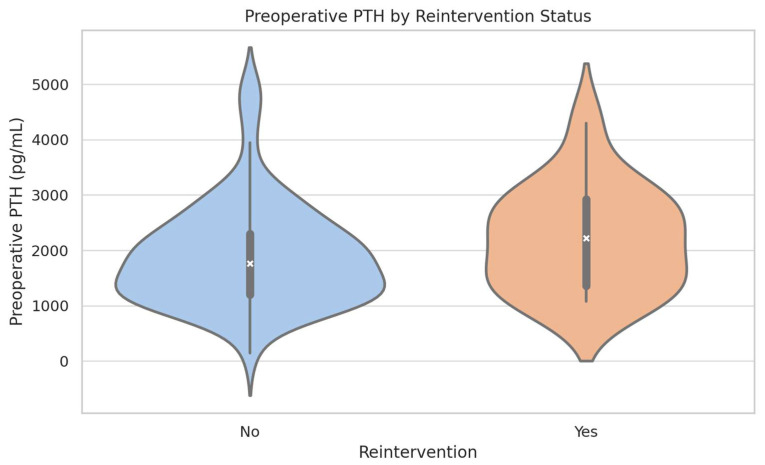
Preoperative Parathyroid Hormone (PTH) Levels as Stratified by Reintervention Status—Violin plot illustrating the distribution and variability of preoperative intact PTH levels among patients who required reintervention for persistent/recurrent hyperparathyroidism (HPT), compared to those who did not. Patients undergoing reintervention typically exhibited higher median PTH levels (2222 pg/mL, IQR: 1355–2925.5 pg/mL) compared to those without reintervention (1767.5 pg/mL, IQR: 1200–2302.25 pg/mL), highlighting the possible predictive importance of elevated preoperative PTH in identifying individuals at greater risk for subsequent interventions (despite not reaching statistical significance—*p* = 0.145).

Regarding the relationship between preoperative parameters and primary postoperative outcomes (i.e., reintervention for persistent/recurrent HPT and HBS), we note that iPTH levels differed significantly between patients who required reintervention and those who did not, with higher median values and greater variability observed among those needing additional surgery (see Figure 2). Specifically, a clear dose–response relationship was observed, with escalating PTH levels associated with increased reintervention rates, peaking notably at levels exceeding 2000 pg/mL (22.5%, *p* = 0.024), as seen in Figure 1B.

Moreover, preoperative serum ALP, as a marker of bone turnover, demonstrated a significant correlation with postoperative complications, particularly HBS. As seen in Figure 1C, the incidence of HBS sharply increased from no observed cases in patients with ALP levels below 150 U/L to universal occurrence (100%) in patients whose preoperative ALP exceeded 300 U/L (*p* < 0.001), underlining the predictive utility of ALP measurements for anticipating postoperative calcium management challenges.

All surgeries resulted in immediate and substantial reductions in iPTH levels. The overall median postoperative Day 1 iPTH was 77.1 pg/mL, reflecting an >95% reduction from preoperative baseline (~1800 pg/mL) on average. Serum calcium dropped from a mean of 10.42 mg/dL pre-op to 7.47 mg/dL on post-op Day 2 despite supplementation, indicating the expected “hungry bone” effect in many patients. Symptomatic improvement in SHPT-related issues was evident: for example, bone pain scores improved by at least one category in 85% of those who had preoperative pain, and the severe pruritus was resolved in all 10 patients.

**Figure 3:**
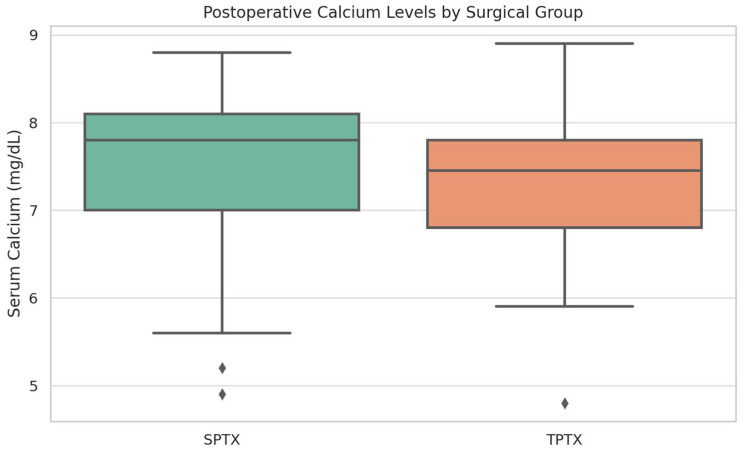
Postoperative Serum Calcium Levels as Stratified by Surgical Approach—Box plot illustrating the postoperative serum calcium levels seen between surgical subgroups. Patients who underwent total parathyroidectomy (TPTX) showed marginally lower postoperative calcium levels (mean 7.32 mg/dL ± 0.80 mg/dL), as compared to subtotal parathyroidectomy (SPTX) cases (mean 7.55 mg/dL ± 0.86 mg/dL).

Serum iPTH was significantly lower postoperatively in the TPTX group compared to SPTX (median 38.7 pg/mL vs. 108.6 pg/mL, *p* < 0.001). Although, as seen in Figure 3, postoperative serum calcium levels were comparable between the two groups (7.3 mg/dL in TPTX vs. 7.6 mg/dL in SPTX, *p* = 0.19), the incidence of HBS was significantly higher among TPTX patients (57.1% vs. 35.3%, *p* = 0.06).

### 3.3. Multivariate Analysis of Primary Postoperative Outcomes

#### 3.3.1. Predictors of Required Reintervention for Persistent/Recurrent HPT

Multivariate logistic regression analysis identified several independent predictors of reintervention for persistent/recurrent hyperparathyroidism (Table 2). The absence of concomitant thymectomy emerged as the strongest risk factor (OR 5.68, 95% CI 1.98–16.32, *p* = 0.001), with no patients who underwent thymectomy requiring reintervention (0% vs. 19.2% without thymectomy). This finding suggests that routine thymectomy during parathyroidectomy may improve long-term disease control, potentially by removing ectopic parathyroid tissue.

The surgical approach was another significant predictor, with SPTX associated with nearly four times higher risk of reintervention compared to TPTX (OR 3.88, 95% CI 1.09–13.84, *p* = 0.037). Preoperative hypercalcemia (>11 mg/dL) independently increased reintervention risk (OR 4.16, 95% CI 1.17–14.79, *p* = 0.028), as did the identification of two or fewer enlarged glands on preoperative imaging (OR 3.12, 95% CI 1.28–7.62, *p* = 0.012) and elevated preoperative PTH levels above 2000 pg/mL (OR 2.83, 95% CI 1.15–6.96, *p* = 0.024).

Patient age showed a slight inverse association with reintervention risk (OR 0.96 per year, 95% CI 0.92-1.01, *p* = 0.089), suggesting a marginally lower risk in older patients, though this did not achieve statistical significance.

#### 3.3.2. Predictors of Hungry Bone Syndrome

In Table 3, within the multivariate logistic regression analysis for predictors of HBS, preoperative ALP >300 U/L emerged as the most potent risk factor (OR 26.53, 95% CI 8.29–84.87, *p* < 0.001). This finding was consistent with our univariate observations, where patients with ALP >300 U/L demonstrated a 100% incidence of HBS compared to 22.7% in those with lower ALP levels. The extreme strength of this association underscores the critical role of high bone turnover in predisposing patients to postoperative hypocalcemia.

Severe bone pain (VAS 7–10) was also independently associated with increased HBS risk (OR 4.60, 95% CI 1.49–14.16, *p* = 0.016), likely reflecting the clinical manifestation of increased bone turnover and mineral hunger. Total parathyroidectomy independently increased HBS risk compared to the subtotal approach (OR 2.83, 95% CI 1.06–7.58, *p* = 0.039).

Preoperative PTH levels demonstrated a small but statistically significant association with HBS development (OR 1.002 per pg/mL increase, 95% CI 1.001–1.003, *p* = 0.031), while osteoporosis showed a positive but non-significant trend (OR 2.05, 95% CI 0.80–5.26, *p* = 0.271). Interestingly, shorter dialysis vintage (≤7 years) showed a positive association with HBS risk (OR 1.58, 95% CI 0.73–3.44, *p* = 0.119), though without achieving statistical significance.

**Figure 4:**
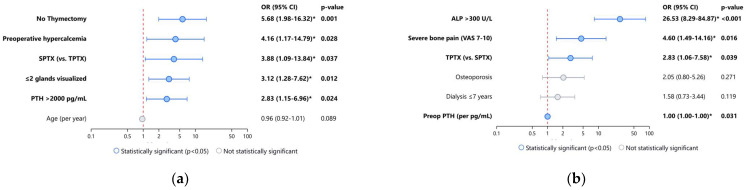
Risk Factors for Surgical Outcomes—Forest plots showing adjusted odds ratios with 95% confidence intervals for variables associated with surgical outcomes in SHPT patients: (**a**) Risk factors for reintervention, demonstrating significant associations for absence of thymectomy, preoperative hypercalcemia, SPTX approach, limited gland visualization, and elevated PTH; (**b**) Risk factors for hungry bone syndrome, showing the strong predictive value of elevated alkaline phosphatase, severe bone pain, TPTX approach, and preoperative PTH levels. Note: OR = Odds Ratio; CI = Confidence Interval; SHPT = Secondary Hyperparathyroidism; SPTX = Subtotal Parathyroidectomy; TPTX = Total Parathyroidectomy; PTH = Parathyroid Hormone; ALP = Alkaline Phosphatase; VAS = Visual Analogue Scale. * *p* < 0.05.

Figure 4 displays forest plots of the adjusted ORs with 95% CIs for predictors of surgical reintervention for persistent/recurrent HPT (Figure 4a) and HBS (Figure 4b), following PTX for SHPT. These visualizations highlight the relative strength of association for each predictor, with the vertical red dashed line indicating the null reference (OR = 1.0). Variables are sorted by descending OR. Asterisks indicate statistical significance (*p* < 0.05).

#### 3.3.3. Subgroup Analyses

To further characterize risk factors for HBS, we conducted additional subgroup analyses. When stratifying patients by preoperative creatinine quartiles, we observed a clear relationship between creatinine levels and HBS incidence. Patients in the lowest creatinine quartile (≤9.80 mg/dL) experienced substantially lower HBS rates (20.8%) compared to those in the highest quartile (>10.39 mg/dL), who had a 65.2% incidence of HBS (*p* = 0.018). This suggests that more severe renal dysfunction may impair the kidney’s ability to compensate for calcium shifts during the immediate postoperative period.

Bone pain severity also demonstrated significant associations with primary outcomes (see Table 4). Patients with severe bone pain (VAS 7–10) had markedly higher rates of HBS (73.7%) compared to those with moderate pain (33.3%, *p* = 0.004). This finding aligns with our multivariate analysis and suggests that severe bone pain may serve as a clinical indicator of increased bone turnover and more severe hyperparathyroid bone disease.

Finally, bone disease status demonstrated associations with both outcomes of interest (see Table 5). Patients with established osteoporosis showed higher rates of both reintervention (20.8% vs. 14.3%) and HBS (58.3% vs. 38.1%) compared to those without bone disease, though these differences did not reach statistical significance (*p* = 0.51 and *p* = 0.09, respectively). The small subgroup of patients with pathological bone fractures (*n* = 2) exhibited a notably high reintervention rate (50.0%), suggesting that severe bone disease may predispose to recurrent hyperparathyroidism, though the limited sample size precludes definitive conclusions.

### 3.4. Correlation Analysis of Key Parameters and Primary Outcomes

**Figure 5:**
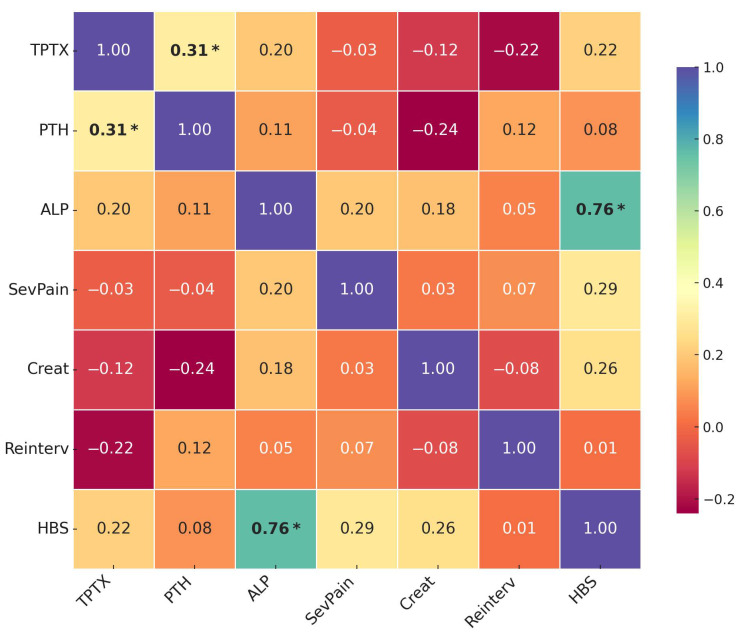
Correlation Matrix of Key Parameters and Primary Outcomes. Note: TPTX = total parathyroidectomy; PTH = preoperative intact parathyroid hormone; ALP = alkaline phosphatase; SevPain = severe bone pain (VAS 7–10); Creat = baseline serum creatinine; Reinterv = reintervention for persistent/recurrent hyperparathyroidism; HBS = hungry bone syndrome. * Correlation is significant at *p* < 0.01.

To further investigate the interrelationships between clinical variables and surgical outcomes, we performed a correlation analysis using Pearson’s correlation coefficient (see Figure 5). This analysis revealed several statistically and clinically significant associations that complement our multivariate regression findings. Only correlations with *p* < 0.01 were considered significant after Bonferroni adjustment for multiple comparisons. Herein, we observed a strong positive correlation between preoperative ALP and the occurrence of HBS (r = 0.76, *p* < 0.001), much higher than any other correlation noted.

#### 3.4.1. Correlations with Reintervention for Persistent/Recurrent Hyperparathyroidism

The correlation analysis confirmed an inverse relationship between TPTX and reintervention risk (r = −0.22), supporting our multivariate findings that TPTX is protective against persistent/recurrent HPT. Preoperative iPTH showed a positive correlation with reintervention (r = 0.12), again aligning with our regression model, where higher PTH was associated with increased reintervention risk. Notably, there was no appreciable correlation between HBS and reintervention (r = 0.01), suggesting that these two major outcomes represent distinct pathophysiological processes rather than being interrelated complications.

#### 3.4.2. Correlations with Hungry Bone Syndrome

There was a strong positive correlation between ALP and HBS (r = 0.76, *p* < 0.001), substantially higher than any other parameter. The strength of this correlation suggests that ALP could serve as a reliable clinical marker for risk stratification and preoperative planning.

TPTX demonstrated a moderate positive correlation with HBS (r = 0.22), supporting our observation that this surgical approach is associated with increased risk of postoperative hypocalcemia. Severe bone pain also showed a notable correlation with HBS development (r = 0.29), reinforcing its potential utility as a clinical predictor of increased bone turnover and calcium demand.

Preoperative serum creatinine exhibited a positive correlation with HBS (r = 0.26), consistent with our subgroup analysis findings that patients with higher creatinine levels had substantially increased HBS rates. This relationship likely reflects reduced capacity for calcium regulation in patients with more advanced renal dysfunction.

#### 3.4.3. Correlations Between Clinical Parameters

There was a moderate positive correlation between TPTX and preoperative PTH levels (r = 0.31, *p* < 0.01), indicating that patients with more severe biochemical derangements were preferentially selected for the more radical surgical approach. This selection bias may partially account for the divergent clinical outcomes between surgical groups and warrants consideration when interpreting comparative efficacy data.

We observed a weak negative correlation between preoperative PTH and creatinine (r = −0.24), suggesting that patients with more advanced renal dysfunction may not always manifest proportionally elevated PTH levels, potentially due to nutritional factors or uremic suppression of parathyroid gland function.

The correlation of ALP with severe bone pain (r = 0.20) and with creatinine (r = 0.18) further supports the complex interrelationships between laboratory parameters, clinical symptoms, and kidney function in SHPT patients.

## 4. Discussion

The management of SHPT in dialysis patients remains complex and demands an individualized approach. Our study provides a comparative evaluation of SPTX versus TPTX, predominantly with AT, addressing crucial postoperative outcomes. Both procedures effectively achieved long-term normal calcemia, phosphate control, and symptom alleviation, confirming prior observations that PTX significantly improves quality of life and clinical outcomes in severe SHPT [4,22,23]. Registry analyses have demonstrated a 15–30% lower mortality rate among dialysis patients undergoing PTX compared to medically treated patients [14,24]. Although our study lacked a non-surgical control group, our cohort’s overall survival supports this PTX clinical benefit hypothesis.

Our findings highlight essential trade-offs between the two surgical techniques. SPTX preserved parathyroid function effectively, eliminating permanent hypoparathyroidism yet resulting in a higher recurrence rate (23.5%), with a substantial subset (12 cases) requiring reintervention. This recurrence risk aligns with prior studies, which consistently report recurrence rates of approximately 10–30% following subtotal surgery [12], with the caveat that degree of specialization and surgical experience will definitely influence these rates by implicitly altering the amplitude of SPTX, i.e., inversely correlating with the degree of PTX tissue removal. Conversely, TPTX almost eliminated recurrence risk (7.1%), at the expense of significantly higher rates of HBS (57.1%) and transient profound hypocalcemia. The increased risk of HBS following TPTX has also been extensively documented, with reported incidences ranging widely from 20% to over 70% [25,26].

Forearm autotransplantation introduces additional considerations, including potential graft site morbidity, recurrent hyperparathyroidism from graft hyperplasia, and graft failure resulting in permanent hypoparathyroidism. Complications such as graft hematoma, infection, persistent pain, and paresthesia at the transplantation site are rare but clinically significant. In our cohort, two patients experienced minor forearm complications: one hematoma resolved conservatively, and one case of transient local numbness. These findings reinforce that while TPTX + AT minimizes cervical re-exploration risk, the forearm site is not complication-free [27].

In line with our findings, Isaksson et al. found that TPTX significantly reduced reintervention rates compared to SPTX, but was associated with higher cardiovascular event rates, presumably due to more pronounced reductions in circulating PTH [17]. Concerns regarding adynamic bone disease (ABD) and increased vascular calcification associated with very low PTH levels post-TPTX remain controversial and deserve further research [19].

Our finding that 88.1% of TPTX patients achieved postoperative iPTH levels below 2× ULN, compared to 66.7% of SPTX patients, raises important concerns about the long-term skeletal consequences of these surgical approaches. While achieving target PTH levels is crucial for controlling mineral metabolism, excessively low PTH levels—particularly those persistently below 2× ULN—have been associated with the development of ABD. Herein, ABD is characterized by markedly reduced bone turnover, impaired fracture healing, and increased fracture risk. The significantly higher proportion of TPTX patients achieving very low iPTH levels in our study (*p* = 0.016) suggests that these patients may be at greater risk for ABD, particularly those without AT. Indeed, the median postoperative iPTH in the TPTX group was only 18.0 pg/mL compared to 67.0 pg/mL in the SPTX group, with some TPTX patients having iPTH levels as low as 0.3 pg/mL. This represents another important trade-off when choosing between surgical approaches: while TPTX virtually eliminates recurrence risk, it may predispose patients to a different but equally concerning skeletal complication. Future studies should include long-term follow-up with bone histomorphometry or alternative markers of bone turnover to better characterize the incidence of ABD following different PTX techniques.

Conversely, there were several independent factors influencing recurrence and HBS risks, aiding surgical decision-making. Notably, the absence of concomitant thymectomy, fewer visualized glands, higher preoperative iPTH, and preoperative hypercalcemia predicted reintervention. Routine thymectomy, although debated, appears justified, as ectopic or supernumerary glands frequently contribute to disease persistence/recurrence [28]. Conversely, high preoperative ALP and severe bone pain, both markers of severe osteitis fibrosa, significantly predicted postoperative HBS [29]. Recognizing these predictors allows targeted preoperative counseling and postoperative management strategies, reducing morbidity and optimizing patient outcomes.

Identifying patient subgroups that might benefit from a specific surgical approach could enhance clinical outcomes. Patients with markedly elevated ALP and severe bone pain, indicating high bone turnover, appear to benefit more from TPTX, given its lower recurrence rates, despite the higher risk of HBS. Conversely, younger patients, patients awaiting transplantation, or those with moderate SHPT may have improved long-term outcomes with SPTX due to preservation of endogenous parathyroid tissue, reducing the risk of permanent hypocalcemia [24].

A key finding is that recurrence and HBS were independent phenomena, not correlated in our analysis. Recurrence primarily results from incomplete parathyroid removal or remnant regrowth, whereas HBS arises from abrupt postoperative calcium influx into severely depleted bones. Therefore, the surgical choice should consider distinct patient profiles rather than treating SHPT uniformly. For instance, transplant candidates might benefit from SPTX, preserving some parathyroid functionality and reducing long-term hypocalcemia risk post-transplantation. Conversely, patients with severe calciphylaxis or extensive osteitis fibrosa cystica may favor TPTX to ensure definitive PTH reduction, despite the risk of transient hypocalcemia [30,31].

Regarding kidney transplantation, tertiary HPT persists in up to 50% of recipients post-transplant, negatively impacting graft survival [32]. Recent evidence suggests that extensive PTX pre-transplantation might reduce tertiary HPT and improve long-term outcomes [33]. Moreover, recent advances in renal tumor immunology and molecular profiling may also contribute to more refined postoperative risk stratification and therapeutic guidance in transplant recipients [34]. These observations highlight the need for individualized surgical planning concerning anticipated transplantation timing and expected postoperative management burdens.

The introduction of calcimimetics initially reduced PTX rates; however, large trials (such as EVOLVE) have not demonstrated survival benefits with these medications [35]. Recent data indicate increased PTX utilization, reflecting recognition of refractory SHPT despite broader calcimimetic availability [30]. Our findings reinforce surgery as essential for definitive SHPT management, underscoring calcimimetics as adjuncts rather than replacements. Although cinacalcet and newer agents (etelcalcetide) effectively manage moderate SHPT, severe refractory cases frequently progress to surgery [35].

Surgeon’s perspective and technical considerations further influence operative choice [36]. Subtotal procedures may be simpler, avoiding graft-related complications, yet reinterventions after cervical surgery pose higher risks of complications such as nerve injury [37]. Conversely, TPTX + AT, despite technical complexity and graft failure risks, facilitates recurrence management, as graft sites (e.g., forearm) are accessible for revision or ablation [38]. Our experience underscores vigilance for supernumerary glands; selective thymectomy during initial surgery may substantially reduce the risk of recurrence.

The present study has several limitations. Its retrospective design and moderate sample size limit conclusions and introduce selection biases influencing procedural choice. Follow-up duration (median ~4 years) might underrepresent late recurrence rates, especially post-SPTX. Moreover, the lack of formal quality-of-life assessments restricts complete evaluation of patient-centered outcomes. Further experimental and translational research using validated animal models, such as the cavernous nerve injury model, may help refine our understanding of postoperative endocrine recovery and nerve-related sequelae [39].

Our data advocate individualized surgical decision-making based on clearly identifiable predictors. Younger patients awaiting transplantation with moderate SHPT may optimally undergo SPTX to preserve parathyroid functionality post-transplant, minimizing hypocalcemia risks. Patients with severe disease, large nodular hyperplastic glands, calciphylaxis, or contraindications to repeat cervical surgery are ideal TPTX candidates. Careful perioperative calcium management mitigates the HBS risk, balancing immediate and long-term benefits [40,41].

Although our study did not include direct economic analysis, it is relevant to consider potential cost implications associated with each surgical strategy. TPTX (particularly with autotransplantation) may incur higher initial costs due to operative time, hospitalization, and calcium management, whereas SPTX, while less costly upfront, may lead to increased long-term costs due to recurrence and potential reintervention. Future cost-effectiveness studies are needed to evaluate these aspects more thoroughly [36].

In summary, our comparative analysis reinforces that SPTX and TPTX both effectively manage severe SHPT but differ significantly in postoperative morbidity profiles. TPTX virtually eliminates recurrence at the expense of transient yet intensive management of HBS, whereas SPTX minimizes hypocalcemia but incurs higher recurrence risks. Stratifying patients by preoperative risk factors such as ALP, PTH level, gland visualization, and transplantation candidacy enables optimized surgical approaches, aligning with modern precision medicine paradigms.

## 5. Conclusions

TPTX significantly reduces recurrence rates compared to SPTX but carries a notably higher risk of HBS, necessitating intensive postoperative calcium management. Preoperative parameters, including markedly elevated ALP (>300 U/L) and severe bone pain, strongly predict the occurrence of HBS, whereas factors such as elevated iPTH, hypercalcemia (>11 mg/dL), fewer visualized glands preoperatively, and absence of concomitant thymectomy significantly predict the need for reintervention. These findings advocate a more tailored, patient-centered approach in surgical decision-making, emphasizing individualized risk stratification.

## Figures and Tables

**Table 1:** Baseline Characteristics of Patients Undergoing TPTX vs. SPTX.

Characteristic	TPTX (*n* = 42)	SPTX (*n* = 51)	*p*-Value
Age (years), mean ± SD	56.8 ± 9.8	50.2 ± 12.2	0.01 *
Sex (male), *n* (%)	24 (57.1)	32 (62.7)	0.74
Dialysis vintage (years), median (IQR)	8.1 (6.0–10.0)	7.0 (4.5–10.0)	0.18
**Preoperative laboratory values**			
Serum calcium (mg/dL), mean ± SD	10.7 ± 1.1	10.2 ± 1.0	0.03 *
Serum intact PTH (pg/mL), median (IQR)	2084 (1500–2730)	1570 (1140–2069)	0.01 *
Alkaline phosphatase (U/L), mean ± SD	281.5 ± 101.2	240.3 ± 97.8	0.05 *
Serum phosphate (mg/dL), mean ± SD	5.49 ± 1.2	5.21 ± 1.1	0.40
Serum creatinine (mg/dL), mean ± SD	9.5 ± 1.9	9.8 ± 1.2	0.24
**Clinical symptoms, *n* (%)**			
Mild bone pain (VAS 1–3)	4 (9.5)	11 (21.6)	0.2
Moderate bone pain (VAS 4–6)	26 (61.9)	28 (54.9)	0.64
Severe bone pain (VAS 7–10)	8 (19.0)	11 (21.6)	0.97
Fatigability	28 (66.7)	32 (62.7)	0.86
Osteoporosis	9 (21.4)	15 (29.4)	0.52
Osteopenia	5 (11.9)	3 (5.9)	0.51
Pathological bone fracture	1 (2.4)	1 (2.0)	1
Renal lithiasis	13 (31.0)	9 (17.6)	0.21
**Preoperative imaging findings**			
Number of enlarged glands, median (IQR)	2.0 (1.0–3.8)	2.0 (1.0–3.0)	0.31

* Statistically significant differences (*p* < 0.05) were determined using Student’s *t*-test (continuous normally distributed data), Mann–Whitney U test (continuous non-normally distributed data), and Chi-square test (categorical data). PTH: Parathyroid hormone; SPTX: subtotal parathyroidectomy; TPTX: total parathyroidectomy; SD: Standard deviation; IQR: Interquartile range; VAS: Visual Analogue Scale.

**Table 2:** Multivariate Logistic Regression Analysis for Prediction of Reintervention Requirement.

Variable	Odds Ratio	95% CI	*p*-Value
No concomitant thymectomy	5.68	1.98–16.32	0.001 *
Preoperative hypercalcemia (>11 mg/dL)	4.16	1.17–14.79	0.028 *
SPTX (vs. TPTX)	3.88	1.09–13.84	0.037 *
≤2 glands visualized	3.12	1.28–7.62	0.012 *
Preoperative PTH >2000 pg/mL	2.83	1.15–6.96	0.024 *
Age (per year)	0.96	0.92–1.01	0.089

* Statistically significant (*p* < 0.05). Multivariate logistic regression model including all variables shown simultaneously. Reintervention was required in 15 patients (16.1% of the cohort), with significantly higher rates in the SPTX group (23.5%) compared to the TPTX group (7.1%). Notably, no patients who underwent concomitant thymectomy required reintervention. Model performance metrics: Nagelkerke R^2^ = 0.436, Hosmer–Lemeshow goodness of fit *p* = 0.85, Area Under the ROC Curve = 0.838 (95% CI: 0.745–0.931).

**Table 3:** Multivariate Logistic Regression Analysis for Prediction of Hungry Bone Syndrome.

Variable	Odds Ratio	95% CI	*p*-Value
Alkaline phosphatase >300 U/L	26.53	8.29–84.87	<0.001 *
Severe bone pain (VAS 7–10)	4.60	1.49–14.16	0.016 *
TPTX (vs. SPTX)	2.83	1.06–7.58	0.039 *
Osteoporosis	2.05	0.80–5.26	0.271
Dialysis ≤7 years	1.58	0.73–3.44	0.119
Preoperative PTH (per pg/mL)	1.002	1.001–1.003	0.031 *

* Statistically significant (*p* < 0.05). Multivariate logistic regression model adjusted for all variables shown in the table. Hungry Bone Syndrome (HBS) occurred in 42 patients (45.2% of the cohort), with significantly higher incidence in the total parathyroidectomy group (57.1%) compared to the subtotal parathyroidectomy group (35.3%). Model performance metrics: Nagelkerke R^2^ = 0.382, Hosmer–Lemeshow goodness of fit *p* = 0.76, Area Under the ROC Curve = 0.801 (95% CI: 0.712–0.890).

**Table 4:** Primary Outcomes by Bone Pain Severity.

Pain Severity	*n*	Reintervention Rate (%)	HBS Rate (%)
None	5	0.0	60.0
Mild (VAS 1–3)	15	20.0	46.7
Moderate (VAS 4–6)	54	14.8	33.3
Severe (VAS 7–10)	19	21.1	73.7 *

* *p* < 0.01 compared to the moderate pain group. VAS = Visual Analogue Scale; *n* = number; HBS = Hungry Bone Syndrome.

**Table 5:** Primary Outcomes by Bone Disease Status.

Bone Disease	*n*	Reintervention Rate (%)	HBS Rate (%)
Osteoporosis	24	20.8	58.3
Osteopenia	8	25.0	50.0
Pathological Fracture	2	50.0	50.0
No Bone Disease	63	14.3	38.1

*n* = number; HBS = Hungry Bone Syndrome.

## Data Availability

Data available on request.

## Abbreviations

The following abbreviations are used in this manuscript:

ALP	Alkaline Phosphatase
AT	Autotransplantation
AUC	Area Under the Curve
CKD	Chronic Kidney Disease
CKD-MBD	Chronic Kidney Disease–Mineral Bone Disorder
ESRD	End-Stage Renal Disease
HBS	Hungry Bone Syndrome
HPT	Hyperparathyroidism
iPTH	Intact Parathyroid Hormone
IQR	Interquartile Range
PTX	Parathyroidectomy
PTH	Parathyroid Hormone
ROC	Receiver Operating Characteristic
RCT	Randomized Clinical Trial
SD	Standard Deviation
SHPT	Secondary Hyperparathyroidism
SPTX	Subtotal Parathyroidectomy
TPTX	Total Parathyroidectomy
VAS	Visual Analogue Scale
